# Contribution of Anti-type IV Collagen Antibodies From Kidney Basement Membrane to Increased Level of Urinary Immunoglobulin G in Nephritis Patients: A Comparison of Nephritic and Healthy Urine

**DOI:** 10.7759/cureus.83284

**Published:** 2025-05-01

**Authors:** Tsukao Yokoyama, Yukisato Ishida

**Affiliations:** 1 Research, Collagen Research Center, Tokyo, JPN; 2 Pharmacology, Tokyo Metropolitan University, Tokyo, JPN

**Keywords:** anti-nc1 antibodies, basement membrane, collagen iv, igg, kidney, nc1, nephritis, urine, α5 chain

## Abstract

Background: Abnormality in the NC1 domain (NC1) of collagen IV is often associated with nephritis. As the α5 chain is common in heterotrimeric collagen IV within kidney basement membranes, in this study, we attempted to elucidate the nephritis-associated alteration of urinary levels of anti-α5 chain NC1 antibodies in relation to those of immunoglobulin G (IgG).

Methods: Urine samples from 19 patients (seven diabetic nephritis, six IgA nephropathy, four nephrotic syndrome, one membranous nephropathy, and one chronic glomerulonephritis) were compared with urine samples from 19 controls (healthy subjects with normal ranges for urinary protein, urinary glucose, urinary occult blood, serum creatinine, serum urea nitrogen, serum uric acid, and estimated glomerular filtration rate (eGFR)) by enzyme-linked immunosorbent assay (ELISA) (A450nm). Using ELISA, antibodies in urine samples were detected by coating the plate with urinary proteins for IgG, purified bovine NC1 for anti-NC1 antibodies (NC1Abs), and a synthesized partial peptide in the α5 chain NC1 (yp08) for anti-yp08 antibodies (yp08Abs).

Results: The levels of all measured antibodies were higher in urine samples from the nephritis group (A450nm (mean ± SD): IgG: 1.154 ± 0.298, NC1Abs: 0.479 ± 0.402, yp08Abs: 0.808 ± 0.569) than from the control group (IgG: 0.728 ± 0.236, NC1Abs: 0.051 ± 0.036, yp08Abs: 0.147 ± 0.107) (p < 0.01). In addition, the proportions of NC1Abs and yp08Abs in urinary IgG were higher in the nephritis group (NC1Abs/IgG: 0.370 ± 0.292, yp08Abs/IgG: 0.627 ± 0.396) than in the control group (NC1Abs/IgG: 0.071 ± 0.044, yp08Abs/IgG: 0.197 ± 0.127) (p < 0.01). In the nephritis group, the level of IgG correlated well with that of NC1Abs and yp08Abs (IgG versus NC1Abs: p < 0.001, r = 0.7754; IgG versus yp08Abs: p < 0.001, r = 0.7860), whereas this correlation was not present in the control group.

Conclusion: Our results showed that NC1Abs and yp08Abs levels are raised in nephritis patients, accounting for the observed increase in IgG levels. Furthermore, antibodies against the α5 chain of collagen IV, which compose the basement membranes of the renal glomeruli and tubules, are responsible for the increased urine levels of IgG in nephritis patients.

## Introduction

The major component of tissue basement membranes is type IV collagen (collagen IV) with an architecture of heterotrimeric coiled-coil chains, where each chain comprises an N-terminus of 7S domain, a central triple-helical domain, and a C-terminus of non-collagenous (NC1) domain [1,2]. In renal tissue basement membranes, α3α4α5 chains are found in the glomeruli, α5α5α6 chains in the renal tubules, and α5α5α6 chains in Bowman's capsule [3-6].

Mal-expression of collagen IV is associated with the basement membrane disease of many organs, including the kidney [7,8]. In nephritis, the urinary excretion of collagen IV has been reported to be increased [9-11]. Abnormality in the NC1 domain of the α3 or α5 chain (α3NC1 or α5NC1) may be associated with the formation of antibodies against α3NC1 or α5NC1 in sera of nephritis patients with Goodpasture's or Alport's syndrome, respectively [7,12]. Immunoglobulins such as IgG and IgA have been reported to be more or less present in human urine [13-17], and the amount of IgG in urine is increased in nephritis patients.

In previous studies, we investigated the role of collagen in the development of nephritis. We prepared the NC1 domain of collagen IV purified from the bovine adrenal cortex and established monkey and rat nephritis models via injection of the NC1 domain [18]. In our immunohistological study, a monoclonal antibody against the NC1 domain, which we prepared, stained only nephritic kidney specimens and not healthy ones, in both human and monkey, human glomeruli, renal tubules, and Bowman's capsule were stained irrespective of nephritis type, and we observed strong staining of the glomeruli and weak staining of the renal tubules in the monkey model [5,15].

In another study, we further confirmed the increased excretion of anti-NC1 domain antibodies (NC1Abs) into the urine of nephritis patients [19]. As the α5 chain is common in heterotrimeric collagen IV in the basement membranes of kidney organelles, as mentioned above, we searched for epitopes in the NC1 domain of the α5 chain by synthesizing 20-amino-acid fragments of the NC1 domain. Several peptides reacted to the nephritic urine, with the strongest reactant being yp08 (PFISR CAVCE APAVV IAVHS) [20,21].

In the present study, we attempted to quantify the levels of IgG, NC1Abs, and anti-yp08 peptide (yp08Abs) antibodies in urine samples of healthy and nephritis subjects to determine the extent to which antibodies against kidney basement membrane collagen IV contribute to the amount of IgG detected in the urine of human subjects.

## Materials and methods

Study subjects

Urine samples from 19 patients with various types of nephritis (seven diabetic nephritis, six IgA nephropathy, four nephrotic syndrome, one membranous nephropathy, and one chronic glomerulonephritis) were provided from the Tsukuba Medical Laboratory of Education and Research Center, Tsukuba, Ibaraki, Japan. To establish a control group, 19 urine samples from healthy subjects (with normal ranges for urinary protein, urinary glucose, urinary occult blood, serum creatinine, serum urea nitrogen, serum uric acid, and estimated glomerular filtration rate (eGFR)) were supplied by the Department of Oral Surgery, Tokyo Dental University Suidobashi Hospital, Chiyodaku, Tokyo, Japan.

Clinical laboratory test data were collected for all samples by each institution. All samples were for the comparison of the two groups, anonymized prior to donation to this study, with consent from each institution. Although the mean age (36 years) of each group was matched, the difference in age range between the nephritis (30-39 years) and control (19-69 years) groups was not considered for data analyses in this study, except for the estimated glomerular filtration rate (eGFR), as the estimation is based on age and other terms (serum creatinine level and gender).

The difference in sex between the number of nephritis (female: 9, male: 10) and the number of control groups (female: 13, male: 6) was not considered for the data analyses in this study, as we did not find the difference between female and male patients for urine levels of protein, IgG, NC1Abs, and yp08Abs in the healthy groups.

Preparation of collagen IV NC1 domain and synthesized NC1-fragmented peptide

The NC1 domain of collagen IV was prepared from bovine renal cortex according to the collagenase digestion method and was used for the detection of anti-NC1Abs. In order to more selectively detect antibodies against the NC1 domain of the α5 chain, we synthesized a peptide with the 20-amino-acid sequence PFISRCAVCEAPAVVIAVHS (106th-125th amino acids of human α5 NC1 domain: Patents of Japan No. 5156997 and US 8420331 B2), hereafter referred to as yp08 [20,21].

Enzyme-linked immunosorbent assay (ELISA) to measure antibodies in urine samples

Considering some relationship between abnormality in the basement membrane and nephritis, we attempted to detect anti-collagen IV antibodies of NC1Abs and yp08Abs liberated into the urine of nephritis and control subjects using ELISA with antigen probes of NC1 and yp08, as collagen IV is a pivotal component of the basement membrane in the kidney. The amount of IgG in the urine samples was also determined for the estimation of pan-antibodies, as the detected IgG might include anti-collagen IV and other kinds of antibodies.

For detecting IgG, urine was directly coated onto the microplate. For detecting NC1Abs or yp08Abs, the antigen of the NC1 domain or the yp08 peptide was first coated onto the plate.

IgG Assay

For the IgG assay, each urine sample was diluted fivefold with phosphate-buffered saline. The diluted urine (100 uL/well) samples were coated on a microplate (NuncTM MaxiSorp Plate, #468667, Thermo Fisher Scientific Japan, Tokyo), and a blocking agent (250 uL/well; WellChampion, #4900A, Kementec, Taastrup, Denmark) was added at room temperature for 15 minutes. Then, the solution was discarded, and the plate was left at room temperature overnight for the solid-phase coating of urinary proteins. The following day, the plate was treated with horseradish peroxidase (HRP)-conjugated anti-human IgG antibody (100 uL/well; #109-036-003, Jackson ImmunoResearch Laboratories, West Grove, PA) for one hour at room temperature. The microplate was then washed and treated with 100 uL of enzyme substrate solution (TMB ONE, Kementec) for 10 minutes. Then, 50 uL of reaction stop solution (1N sulfuric acid) was added to each well, and absorbance (A450) was measured at a wavelength of 450 nm using a microplate reader (model 680, Bio-Rad, Hercules, CA).

Measurements of NC1Abs

For the NC1Abs measurements, NC1 (5 ug/mL) was applied to a microplate (100 uL/well; NuncTM MaxiSorp Plate), which was left overnight at 4°C. The following day, the plate was treated with a blocking agent (250 uL/well; WellChampion) for 15 minutes at room temperature. Then, the solution was discarded, and the plate was left at room temperature overnight. The following day, 100 uL of fivefold-diluted urine was incubated for two hours at room temperature, and 100 uL of HRP-conjugated anti-human IgG antibody (#109-036-003, Jackson) was incubated for one hour at room temperature. Then, 100 uL of enzyme substrate solution (TMB ONE, #4380H) was incubated for 10 minutes before adding 50 uL of reaction stop solution (1N sulfuric acid). Thereafter, A450 was measured using a microplate reader (Model 680, Bio-Rad).

Measurements of yp08Abs

For the yp08Abs measurements, the same procedure as that for the measurement of NC1Abs was carried out, with peptide yp08 (5 ug/mL) used instead of NC1.

Processing of the obtained results

Based on the assumption that IgG detected in urine includes all kinds of IgG, including NC1Abs and yp08Abs, using the original amounts of measured antibodies, the obtained data were further processed by dividing the amount of NC1Abs or yp08Abs by that of IgG to estimate the proportion of NC1Abs or yp08Abs in IgG, and to know the relative amount of NC1Abs or yp08Abs to that of IgG.

Statistical analysis

Statistical analysis was performed as follows. The measured values ​​(A450nm) of the three items (IgG, NC1Abs, and yp08Abs) were tested for normal distribution in both nephritis and healthy subjects. Normal distribution was not obtained for IgG in nephritis and NC1Abs in healthy subjects, so a nonparametric Mann-Whitney test was performed to compare nephritis and healthy subjects for IgG and NC1Abs.

An F test was performed for yp08Abs between nephritis and healthy subjects, which were normally distributed, and because the variances between nephritis and healthy subjects were not equal, a parametric Welch's t-test was used to compare nephritis and healthy subjects.

The eGFR was normally distributed in both nephritis and healthy subjects, and an F-test was performed. Because the variances between nephritis and healthy subjects were not equal, the parametric Welch's t-test was used to compare nephritis and healthy subjects.

When calculating correlation, Spearman's rank correlation coefficient test was used when including nephritic IgG or NC1Abs from healthy subjects, for which normality could not be obtained, and Pearson's correlation coefficient test was used otherwise.

Statistical significance was set at p < 0.05 unless otherwise stated.

## Results

Clinical features of nephritis and healthy (control) subjects

Table 1 shows the clinical laboratory test results of the urine and blood from the nephritis group and the control group. The test results and urine samples were supplied by each institution. In the control group, the urine samples were negative for urine protein, uric blood, and urine sugar and exhibited normal levels of tested serum items and eGFR (over 60 mL/minute/1.73 m^2^, range: 68-135). In the nephritis group with 19 patients, 12 (63.2%), 10 (52.6%), and four (21.1%) patients were positive for urine protein, uric blood (including four pseudo-positive), and urine sugar, respectively.

**Table 1:** Clinical laboratory test profiles of healthy (control) and nephritis groups Values of age, eGFR, or blood test: mean ± SD (number). p-values between the control and nephritis groups were obtained using the F-test for differences in variance, followed by t-tests for differences in mean value. p-values are provided only when p < 0.05 (one-sided in italics), as shown above. eGFR: estimated glomerular filtration rate, BUN: blood urea nitrogen, HDL: high-density lipoprotein, LDL: low-density lipoprotein, SD: standard deviation

Comparison item	Control group	Nephritis group	p-value
Subject number (female:male)	19 (13:6)	19 (9:10)	-
Age (years)	36.0 ± 14.5 (19)	36.0 ± 2.85 (19)	-
Urine test			
Urinary protein (detected number, ≥±)	0	12	-
Urinary blood (detected number, ≥±)	0	10	-
Urine sugar (detected number, ≥1+)	0	4	-
Kidney filtration function			
eGFR (mL/minute/1.73 m^2^)	91.7 ± 15.7 (19)	72.9 ± 33.0 (19)	<0.05
Blood test (serum)			
Creatinine (mg/dL)	0.67 ± 0.12 (19)	1.25 ± 1.23 (19)	<0.05
BUN (mg/dL)	11.8 ± 2.47 (19)	22.2 ± 23.2 (18)	<0.05
Uric acid (mg/dL)	5.23 ± 1.10 (19)	5.98 ± 1.27 (18)	<0.05
Glucose (mg/dL)	91.0 ± 12.2 (19)	140 ± 59.7 (13)	<0.05
Total cholesterol (mg/dL)	189 ± 31.8 (19)	202 ± 29.2 (12)	-
HDL cholesterol (mg/dL)	63.9 ± 10.8 (14)	61.4 ± 20.1 (12)	-
LDL cholesterol (mg/dL)	96.9 ± 27.7 (14)	105 ± 36.5 (9)	-
Triglyceride (mg/dL)	87.6 ± 48.8 (14)	169 ± 142 (13)	<0.05
Albumin (g/dL)	4.48 ± 0.34 (19)	4.00 ± 0.80 (14)	<0.05

The mean value of eGFR was significantly lower in the nephritis group (72.9 ± 33.0 mL/minute/1.73 m^2^; range: 7.5-126) than in the control group (91.7 ± 15.7 mL/minute/1.73 m^2^; range: 68-135).

Since any single clinical laboratory test could not correctly diagnose all patients with the disease, the test values fall into true positives and false positives in the patient group. The sensitivity (it is positive rate among patients) to identify true positives in the patient group is defined by the following equation: true positives / (true positives + false negatives) × 100 [22]. Thus, the sensitivities (positives / all patients) for the diagnosis of nephritis patients were 63.2 for uric protein, 52.6 for uric blood, 31.6 for eGFR (<60 mL/minute/1.73 m^2^), 52.6 for serum creatinine, 22.2 for serum BUN, 44.4 for serum uric acid, and 28.6 for serum albumin. The reason for some sample numbers of parameters being <19 was unknown, as the data were supplied from Tsukuba University Hospitals.

**Figure 1:**
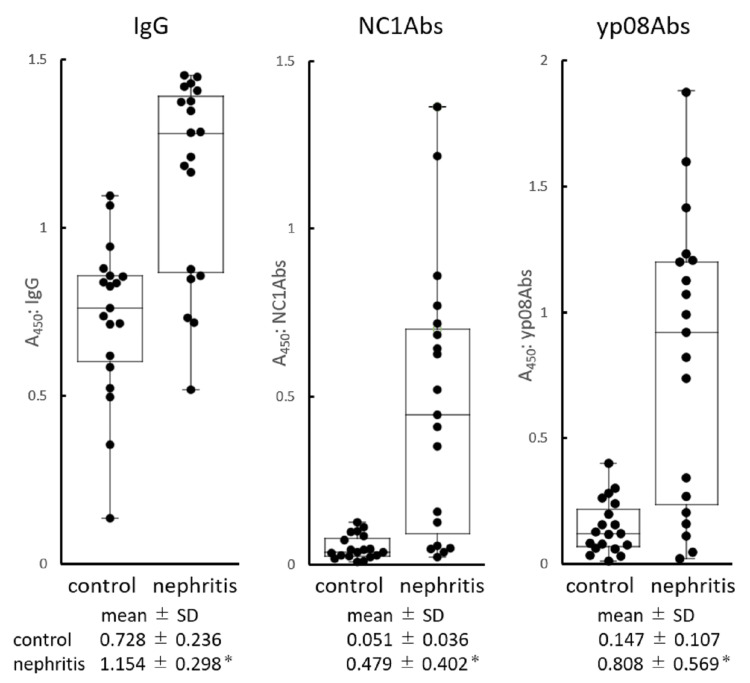
Rank order Rank order of amounts (A450) of IgG, NC1Abs, and anti-yp08Abs in urine samples from healthy (control) and nephritis groups. Values of mean A450 ± standard deviation are shown below each plot for control and nephritis groups, where * indicates p < 0.001 (n = 19 each). The box shows the median and interquartile range. IgG: immunoglobulin G, NC1Abs: anti-NC1 antibodies, yp08Abs: yp08 antibodies, SD: standard deviation

Figure 1 shows the A450 values of IgG, NC1Abs, and yp08Abs detected in urine samples from the control and nephritis groups. These tested antibodies were detected in all urine samples collected to varying extents. The mean A450 values of the detected antibodies were significantly elevated in the nephritis group as compared with the control group. Specifically, the mean A450 values of IgG, NC1Abs, and yp08Abs were 1.6, 9.4, and 5.5 times higher in the nephritis group than in the control group, respectively. Notably, the A450 values of anti-collagen IV antibodies had a narrower distribution range in the control group than in the nephritis group, leading to higher nephritis-to-control A450 ratios. The sensitivities for the diagnosis of nephritis were 10.5 for IgG, 63.2 for NC1Abs, and 63.2 for yp08Abs. Patients with more than the control mean A450nm ± 3 SD were considered positive. Sensitivity is the number of positives divided by the number of people with nephritis.

Relative amounts of NC1Abs and yp08Abs to IgG in urine samples of control and nephritis groups

**Figure 2:**
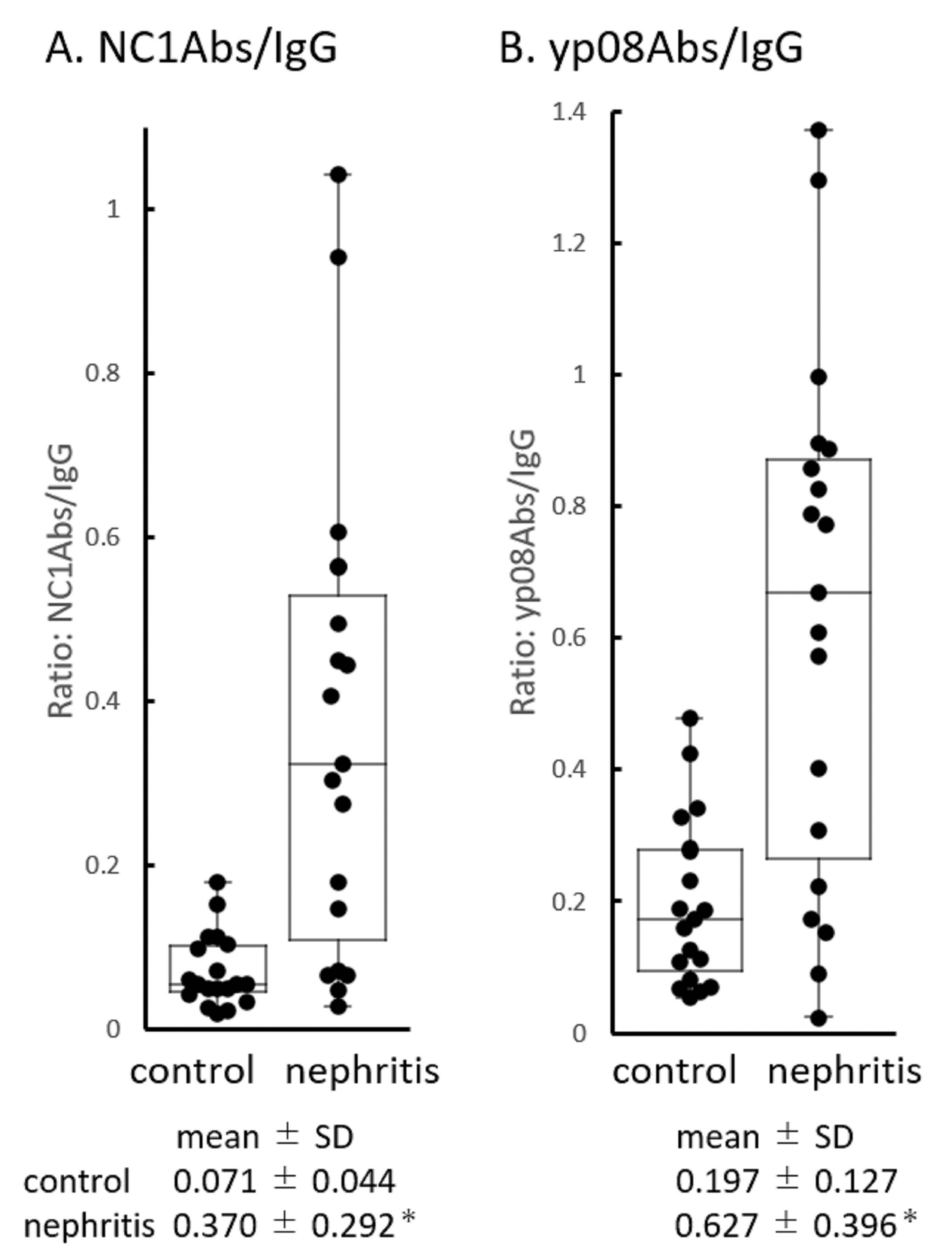
Rank order of normalized amounts Rank order of normalized amounts of NC1Abs (A) and yp08Abs (B) based on the amount of IgG in urine samples from control and nephritis subjects. Ordinate: A450 ratio of NC1Abs to IgG (A) or yp08Abs to IgG (B). Values of mean ± SD for control and nephritis groups are shown below each plot, where * indicates p < 0.001. The box shows the median and interquartile range. IgG: immunoglobulin G, NC1Abs: anti-NC1 antibodies, yp08Abs: yp08 antibodies, SD: standard deviation

To understand the extent to which anti-collagen IV antibody level contributes to that of IgG, the A450 value of NC1Abs or yp08Abs was divided by that of IgG. Figure 2 shows the A450 ratio of NC1Abs or yp08Abs to IgG for each urine sample from the nephritis and control groups. In the control group, the mean ratios were relatively low: 0.071 and 0.197 for NC1Abs/IgG and yp08Abs/IgG, respectively. The mean ratios of NC1Abs/IgG and yp08Abs/IgG were 5.2 and 3.2 times greater in the nephritis group (A450 ratio ranges: 0.027-1.044 for NC1Abs/IgG and 0.024-1.372 for yp08Abs/IgG) than in the control group (A450 ratio ranges: 0.019-0.179 for NC1Abs/IgG and 0.053-0.478 for yp08Abs/IgG), respectively.

Correlation between A450 values of NC1Abs and yp08Abs

**Figure 3:**
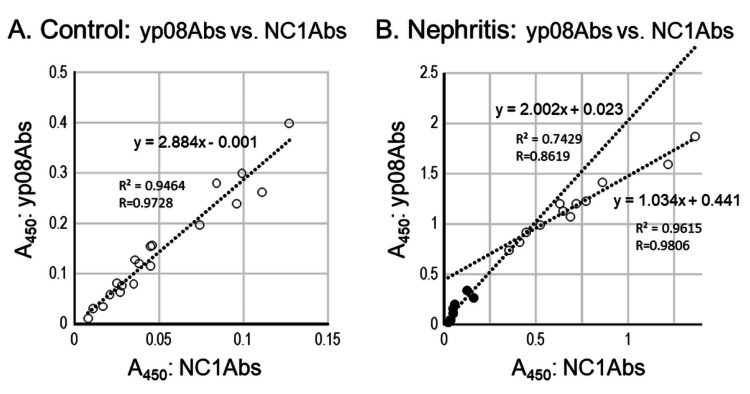
Relationship Relationship between NC1Abs and yp08Abs levels in control (A) and nephritis (B) urine samples. Abscissa: A450 of NC1Abs. Ordinate: A450 of yp08Abs. In B, two-component analysis is employed by dividing groups at 0.5 A450 of yp08Abs: samples with an A450 of <0.5 are represented by closed circles. The dashed lines represent linear regression lines. NC1Abs: anti-NC1 antibodies, yp08Abs: yp08 antibodies

The correlation between A450 values of anti-collagen IV antibodies was analyzed by plotting yp08Abs A450 values against NC1Abs A450 values (Figure 3). In the control urine samples, the plot yielded a linear regression line (Figure 3A): y = 2.884x - 0.001, with a correlation coefficient of r = 0.9728 (y and x; A450 of yp08Abs and NC1Abs, respectively).

In urine samples from the nephritis group, for the linear fitting analysis, the correlation seemed to have at least two components derived from lower and higher A450 values (Figure 3B): in the lower range (<0.2 in NC1Abs and <0.4 in yp08Abs), the linear regression line was y = 2.002x + 0.023 (r = 0.862, n = 7), and in the higher range (>0.3 in NC1Abs and >0.7 in yp08Abs), the linear regression line was y = 1.033x + 0.441 (r = 0.981, n = 12).

In the lower A450 range, the slopes of the regression lines were between 2 and 3 for the control group and seven nephritis subjects, suggesting that the antigen, yp08, captured antibodies at a rate that was approximately 2-3 times more than that of NC1. In contrast, the slope for the higher A450 range was nearly 1 for the rest of the nephritis group, suggesting the comparable ability of NC1 and yp08 antigens to capture antibodies in higher amounts.

Relationship between NC1Abs or yp08Abs and IgG

**Figure 4:**
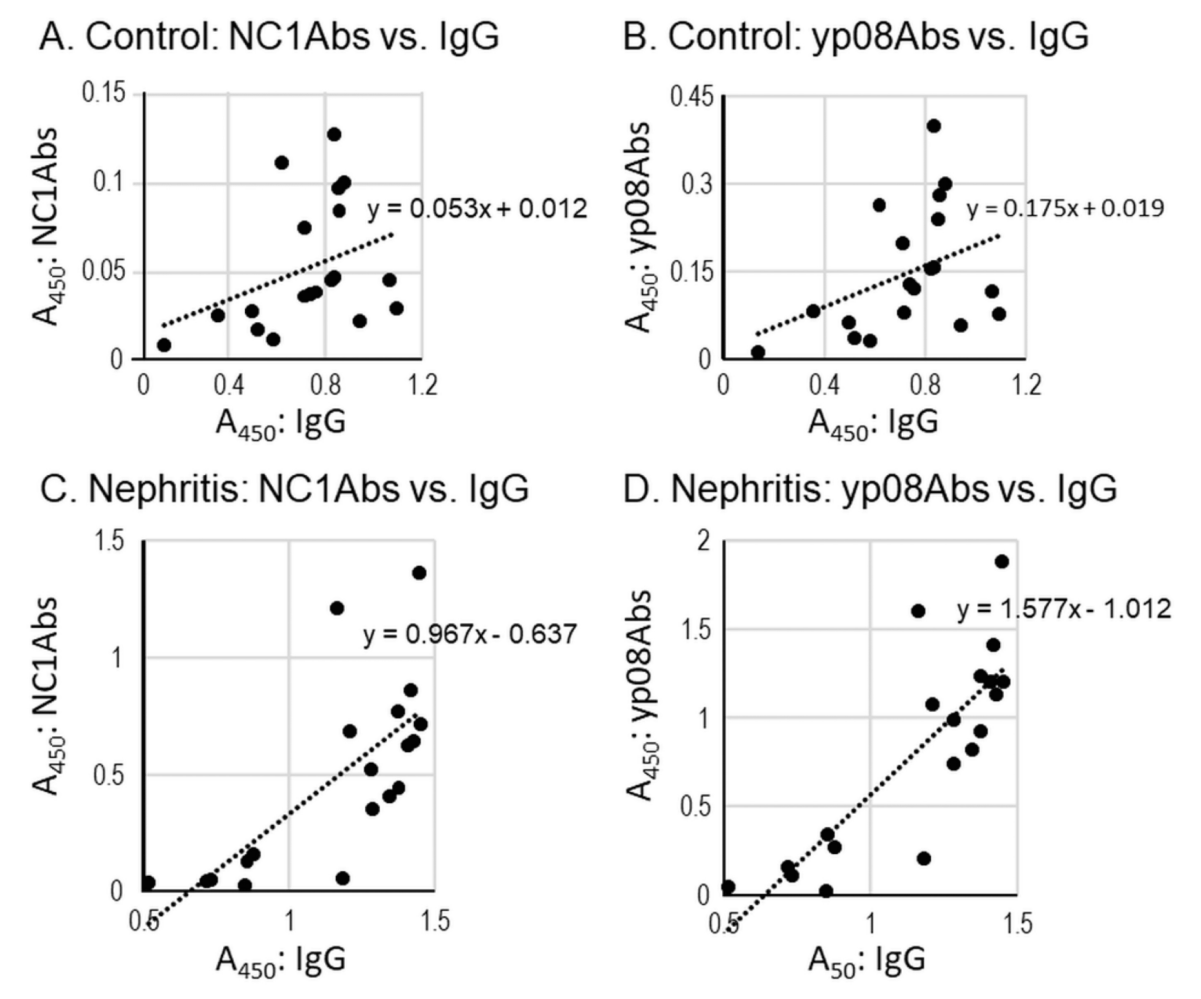
Relationship Relationship between amounts of IgG and NC1Abs (A and C) or yp08Abs (B and D) in control (A and B) and nephritis (C and D) samples. IgG: immunoglobulin G, NC1Abs: anti-NC1 antibodies, yp08Abs: yp08 antibodies

When the A450 of NC1Abs or yp08Abs was plotted against that of IgG, the linear relationship was relatively low in the control urine samples (r = 0.4398 and 0.3882 for NC1Abs versus IgG and yp08Abs versus IgG, respectively) (Figure 4A and Figure 4B) compared to that in the nephritis samples (r = 0.7754 and 0.7860 for NC1Abs versus IgG and yp08Abs versus IgG respectively) (Figure 4C and Figure 4D).

## Discussion

The presence of urinary IgG, responsible for many kinds of antigens, has been reported in many diseases [23]. Here, the relationships between IgG and anti-collagen antibodies detected in the urine are discussed.

In the present investigation, antibodies were more or less detected as IgG, NC1Abs, and yp08Abs in urine samples collected from both control and nephritis subjects. The presence of small amounts of antibodies even in healthy subjects suggests that many kinds of antibodies are continuously produced to maintain the body's normal general function. In kidney diseases, the turnover rate of collagen IV was reported to be augmented [24].

Based on the ELISA results, the mean levels of these detected antibodies were elevated in nephritic urine samples when compared with control samples, and this elevation was much greater for NC1Abs and yp08Abs than for IgG. The level ratios of NC1Abs and yp08Abs to IgG were apparently increased in the nephritic samples, as compared with the control samples. These results suggest that the ratio of anti-collagen IV antibodies to total antibodies in the urine is raised in nephritis patients. Therefore, diagnosis for nephritis may be more apparent when using urinary levels of NC1Abs and yp08Abs compared to IgG. However, the sensing degree of IgG, NC1Abs, or yp08Abs level to identify true positives [22] for nephritis was nearly same at 63%-68% of tested samples, which was higher than that (<60%) in laboratory serum and functional tests, with the exception of the urine protein test (63%).

The origin of the anti-collagen IV antibodies detected in this study might be mainly the basement membranes of the kidney, as our previous study showed that the anti-NC1 monoclonal antibody produced by us stained only injured kidneys and not healthy kidneys. The strong staining at the glomeruli and weak staining at the tubules in monkey nephritis models, as well as the staining at the glomeruli, tubules, and Bowman's capsule in human nephritis patients, irrespective of nephritis type [19], may be corroborated with the presence of anti-glomerular basement membrane antibodies associated with progressive renal inflammation. However, the reported epitope resides in the NC1 domain of the α3 chain of collagen IV [25].

In this study, a linear relationship was observed between detected amounts of NC1Abs and yp08Abs in urine samples from control and nephritis subjects. However, the slope of the regression line was 2-3 at small antibody levels for control and nephritis samples and nearly 1 at large antibody levels only in nephritis samples. Although the reason for the difference in slopes between small and large antibody levels is unknown at this moment, the affinity of antigen to capture antibodies may be 2-3 times higher in yp08 than in NC1, or the amount of antigen on the measurement plate was much more in yp08 than in alpha5 NC1 (20-amino-acid peptide in α5 NC1 domain of yp08 and approximately 230-amino-acid peptide in α5 NC1).

In addition, epitopes for the anti-collagen IV α5 chain could be altered by the progression of nephritis, as nephritis brings about a new epitope with higher affinity toward the NC1 domain in addition to the yp08 region. The alteration of epitopes was also reported in anti-glomerular antibody diseases [26-28].

## Conclusions

The concentrations of urine IgG, NC1Abs, and yp08Abs are higher in nephritis patients than in healthy individuals. In the urine of nephritis patients, the proportions of NC1Abs and yp08Abs in IgG increase. Increased amounts of NC1Abs and yp08Abs in the urine may indicate inflammation of the renal basement membrane.
